# Factor Uniqueness of the Structural Parafac Model

**DOI:** 10.1007/s11336-020-09715-4

**Published:** 2020-08-16

**Authors:** Paolo Giordani, Roberto Rocci, Giuseppe Bove

**Affiliations:** 1 Department of Statistical Sciences, Sapienza Università di Roma, P.le Aldo Moro, 5, 00185 Rome, Italy; 2 Sapienza Università di Roma, Rome, Italy; 3 University of Roma Tre, Rome, Italy

**Keywords:** three-way factor analysis, maximum likelihood, factor uniqueness property

## Abstract

Factor analysis is a well-known method for describing the covariance structure among a set of manifest variables through a limited number of unobserved factors. When the observed variables are collected at various occasions on the same statistical units, the data have a three-way structure and standard factor analysis may fail. To overcome these limitations, three-way models, such as the Parafac model, can be adopted. It is often seen as an extension of principal component analysis able to discover unique latent components. The structural version, i.e., as a reparameterization of the covariance matrix, has been also formulated but rarely investigated. In this article, such a formulation is studied by discussing under what conditions factor uniqueness is preserved. It is shown that, under mild conditions, such a property holds even if the specific factors are assumed to be within-variable, or within-occasion, correlated and the model is modified to become scale invariant.

## Introduction

Factor analysis (FA) (Bartholomew, Knott, & Moustaki, 2011) is a well-known method explaining the relationships among a set of manifest variables, observed on a sample of statistical units, in terms of a limited number of latent variables. In FA, data are stored in a matrix, say **X**, of order ($I \times J$) being *I* and *J* the number of statistical units and variables, respectively. Thus, FA deals with two-way two-mode data, where the modes are the entities of the data matrix, i.e., statistical units and manifest variables, and the ways are the indexes of the elements of **X**, i.e., $i = 1, \ldots , I$ and $j = 1, \ldots , J$. In many practical situations, it may occur that the scores on the same manifest variables with respect to a sample of statistical units are replicated across *K* different occasions, e.g., time, locations, conditions, etc. Examples can be found in several domains. In medicine, these are the daily measures of some vital characteristics on a set of patients. In chemistry, samples measured at different emission wavelengths and excitation wavelengths. In social sciences, think about the yearly evaluation of the main cities of a given area in terms of indicators assessing the quality of life. In marketing, ratings on some goods expressed by a sample of consumers. In psychology, the scores for a group of children on a set of personality variables (traits) rated by different judges (methods). In the previous examples, there are three sets of entities (statistical units, manifest variables and occasions), hence three modes. For instance, in the latter one, these are children, traits and methods. The available information is stored in the so-called array, or tensor, usually denoted by ${\underline{\mathbf{X }}}$ of order ($I \times J \times K$). Its generic element is $x_{ijk}$, $i = 1, \ldots , I, j = 1, \ldots , J$ and $k = 1, \ldots , K$, expressing the score of statistical unit *i* (e.g., child) on manifest variable *j* (e.g., trait) at occasion *k* (e.g., method). Therefore, the elements have three indexes and the array three ways. For all of these reasons, data are three-way three-mode. For further details, refer to, e.g., Kiers (2000) and Kroonenberg (2008).

The basic FA model is not adequate to handle three-way three-mode data. The reason is that FA does not take into account properly the multiway multimode structure of the data. In principle, FA could still be applied. Specifically, it is possible to convert ${\underline{\mathbf{X }}}$ into a matrix by the so-called matricization or unfolding. For instance, one can easily obtain the matrix $\mathbf{X }_{\mathrm {A}}$ of order ($I \times \textit{JK}$) by juxtaposing next to each other the frontal slabs of ${\underline{\mathbf{X }}}$ (statistical unit-mode matricization), where the frontal slabs of ${\underline{\mathbf{X }}}$ are the standard two-way two-mode matrices $\mathbf{X }_{k}$ ($k = 1, \ldots , K$) of order ($I \times J$) collected at the different occasions. Nonetheless, the FA model fitted to $\mathbf{X }_{\mathrm {A}}$ discovers latent variables ignoring that the same manifest variables are replicated across the occasions. For this reason, the standard FA model has been extended in order to take into account and exploit the increasing complexity of three-way three-mode data.

The most famous three-way three-mode extensions of FA are essentially based on the three-mode factor analysis model (Tucker, 1963; Tucker, 1966), usually named Tucker3, and parallel factor analysis model (Harshman, 1970), usually named Parafac. The latter can be seen as a particular case of the former with a useful property of parameter uniqueness (Kruskal, 1977). Although such extensions were presented as suitable generalizations of FA, they can be considered as extensions of the principal component analysis (PCA) solution to the FA model. As a matter of fact, Carroll & Chang (1970) proposed a canonical decomposition equivalent to Parafac, named Candecomp; as an extension of PCA in multidimensional scaling, Kroonenberg & De Leeuw (1980) named three-mode principal component analysis a method based on the ordinary least squares (OLS) estimation of Tucker3. For these reasons, hereinafter such models will be said to follow a component-based approach. The reader interested in the relationships between FA and PCA may refer to, for instance, Unkel & Trendafilov (2010) and Adachi & Trendafilov (2019).

Some authors revised Tucker3 and Parafac as structural models for the covariance structure of the manifest variables (see, for example, Bentler, Poon, & Lee, 1988 and references therein). In other terms, the data generation process is explicitly specified. The *I* statistical units are considered independent observations of the same random variable, and the models are reformulated as a suitable reparameterization of the parameters of its distribution. In particular, each model specifies a certain structure for the variables $\times$ occasions covariance matrix where the covariance matrix of the specific factors, i.e., the error term, is explicitly considered (see, for example, Kroonenberg & Oort, 2003).

As far as we know, in the literature, the component-based approach has received much more attention than the structural one. In this paper, after recalling the main features of methods and models characterizing the two approaches, a structural extension of Parafac is considered. It will be shown how its fundamental factor uniqueness property is preserved even when some specific factors are correlated across occasions, or variables, and/or its structure is modified to become scale invariant. The effectiveness of the proposal is illustrated by a real-life example.

## The Component-Based Approach

The Tucker3 model (Tucker, 1966) summarizes the three-way three-mode tensor ${\underline{\mathbf{X }}}$ by looking for a limited number of components for the modes. The matrix formulation of the Tucker3 model in terms of the statistical unit-mode matricization $\mathbf{X }_{\mathrm {A}}$ is

1 $$\begin{aligned} \mathbf{X }_{\mathrm {A}} = \mathbf{AG }_{\mathrm {A}} (\mathbf{C} \otimes \mathbf{B} )^{\prime } + \mathbf{E }_{\mathrm {A}}, \end{aligned}$$

where the symbol ‘$\otimes$’ denotes the Kronecker product of matrices and **A**, **B**, **C** have order ($I \times P$), ($J \times Q$), ($K \times R$), respectively. The Tucker3 model can be seen as a generalization of PCA where the *JK* columns of $\mathbf{X }_{\mathrm {A}}$, *J* variables measured at *K* different occasions, are modeled through the linear combinations of *P* latent components for the statistical units (the columns of **A**), with weights $\mathbf{G }_{\mathrm {A}} (\mathbf{C} \otimes \mathbf{B} )^{\prime }$. By exploiting the symmetry of the model with respect to the modes, we derive that *P*, *Q* and *R* are the number of components for the statistical units, the manifest variables and the occasions, respectively, while the elements of **A**, **B** and **C** are the scores of the entities of the various modes on the corresponding components. Finally, $\mathbf{G }_{\mathrm {A}}$ is the statistical unit-mode matricization, of order ($P \times {\textit{QR}}$), of the so-called core tensor ${\underline{\mathbf{G }}}$, of order ($P \times Q \times R$), expressing the strength of the triple interactions among the components of the three modes. Like PCA, the model parameters are estimated in the OLS sense by minimizing $\Vert \mathbf{E }_{\mathrm {A}}\Vert ^{2}$, where $\Vert \cdot \Vert$ is the Frobenius norm of matrices. The solutions are not obtained by the singular value decomposition (SVD, Eckart & Young, 1936) of a particular matrix as is for PCA. For this purpose, alternating least squares (ALS) algorithms can be applied. See, for instance, Kroonenberg & De Leeuw (1980). As a possible extension of SVD to the multiway case, we mention the higher-order singular value decomposition (De Lathauwer, De Moor, & Vandewalle, 2000).

The applicability of the Tucker3 model may be limited for several reasons. First of all, the interpretability of the solution, in particular of the elements of the core tensor, is usually a very complex issue. Moreover, differently from standard PCA, the solutions are not nested. Finally, the obtained solution is not unique. In fact, it is straightforward to see that we can post-multiply **A**, **B** and **C** by square non-singular transformation matrices of appropriate order, say **P**, **Q** and **R**, obtaining the new component matrices $\mathbf{A }_{\mathrm {T}} = \mathbf{AP }$, $\mathbf{B }_{\mathrm {T}} = \mathbf{BQ }$ and $\mathbf{C }_{\mathrm {T}} = \mathbf{CR }$. If we compensate such transformations in the core by setting $\mathbf{G }_{\mathrm {AT}} = \mathbf{P }^{-1} \mathbf{G }_{\mathrm {A}} (\mathbf{R }^{-1}\otimes \mathbf{Q }^{-1})^{\prime }$, we get an equally well-fitting solution because

2 $$\begin{aligned} \mathbf{A }_{\mathrm {T}} \mathbf{G }_{\mathrm {AT}} (\mathbf{C }_{\mathrm {T}}\otimes \mathbf{B }_{\mathrm {T}})^{\prime } = \mathbf{APP }^{-1} \mathbf{G }_{\mathrm {A}} (\mathbf{R }^{-1}\otimes \mathbf{Q }^{-1}) ^{\prime } (\mathbf{R} \otimes \mathbf{Q} )^{\prime } (\mathbf{C} \otimes \mathbf{B} )^{\prime } = \mathbf{AG }_{\mathrm {A}} (\mathbf{C} \otimes \mathbf{B} )^{\prime }. \end{aligned}$$

To overcome the possible limitations of the Tucker3 model, it may be convenient to consider the Parafac one, independently proposed by Carroll & Chang (1970) and Harshman (1970). The Parafac model can be seen as a constrained version of the Tucker3 one where the same number of components, say *S*, is sought for all the modes ($P = Q = R = S$) and the core tensor is equal to the identity tensor (${\underline{\mathbf{G }}} = {\underline{\mathbf{I }}}$, i.e., $g_{pqr} = 1$, if $p = q = r$, and $g_{pqr} = 0$, otherwise). We have

3 $$\begin{aligned} \mathbf{X }_{\mathrm {A}} = \mathbf{AI }_{\mathrm {A}} (\mathbf{C} \otimes \mathbf{B} )^{\prime } + \mathbf{E }_{\mathrm {A}} = \mathbf{A} (\mathbf{C} \bullet \mathbf{B} )^{\prime } + \mathbf{E }_{\mathrm {A}}, \end{aligned}$$

where the symbol ‘$\bullet$’ denotes the Khatri–Rao product of matrices, i.e., it is $\mathbf{C} \bullet \mathbf{B} = [\mathbf{c }_{1}\otimes \mathbf{b }_{1}, \ldots , \mathbf{c }_{S}\otimes \mathbf{b }_{S}$], where $\mathbf{b }_{s}$ and $\mathbf{c }_{s}$ are the *s*th columns of **B** and **C**, respectively ($s = 1, \ldots , S$). As for the Tucker3 case, the parameter estimates are found in the OLS sense by ALS algorithms.

The most interesting feature of Parafac is that under mild conditions, the solution is unique. This point has been deeply investigated by Kruskal (1977), who has found the following result. Let us denote by *k*-rank(**Z**) the so-called *k*-rank of a matrix **Z**. It is defined as the largest number *k* such that every subset of *k* columns of **Z** is linearly independent. Moreover, let (**A**, **B**, **C**) and ($\mathbf{A }_{\mathrm {T}}$, $\mathbf{B }_{\mathrm {T}}$, $\mathbf{C }_{\mathrm {T}})$ be two Parafac solutions. Kruskal (1977) has shown that if

4 $$\begin{aligned} k\hbox {-rank}(\mathbf{A }) + k\hbox {-rank}(\mathbf{B }) + k\hbox {-rank}(\mathbf{C }) \ge 2S + 2 \end{aligned}$$

then, by considering (3),

5 $$\begin{aligned} \mathbf{A }(\mathbf{C } \bullet \mathbf{B })^{\prime } = \mathbf{A }_{\mathrm {T}} (\mathbf{C }_{\mathrm {T}} \bullet \mathbf{B }_{\mathrm {T}})^{\prime } \end{aligned}$$

implies that there exist a permutation matrix **P** and three diagonal matrices $\mathbf{D }_{\mathrm {A}}$, $\mathbf{D }_{\mathrm {B}}$ and $\mathbf{D }_{\mathrm {C}}$, for which $\mathbf{D }_{\mathrm {A}} \mathbf{D }_{\mathrm {B}} \mathbf{D }_{\mathrm {C}} = \mathbf{I}$, where **I** denotes the identity matrix, such that

6 $$\begin{aligned} \mathbf{A }_{\mathrm {T}} = \mathbf{APD }_{\mathrm {A}}, \mathbf{B }_{\mathrm {T}} = \mathbf{BPD }_{\mathrm {B}}, \mathbf{C }_{\mathrm {T}} = \mathbf{CPD }_{\mathrm {C}}. \end{aligned}$$

In other words, if (4) holds, then the solution (**A**, **B**, **C**) is unique up to scaling and a simultaneous column permutation. Although Kruskal’s condition has been extended by some other authors (Jiang & Sidiropoulos, 2004; Stegeman, ten Berge, & De Lathauwer, 2006; Stegeman, 2009; Domanov & De Lathauver, 2013a; Domanov & De Lathauver, 2013b), what follows is based on such a condition because practitioners mainly refer to it in their applications. In particular, without loss of generality, we set the scaling of the factor loading matrices by assuming that **B** and **C** are column-wise normalized, i.e.,

7 $$\begin{aligned} (\mathbf{B} ^{\prime } \mathbf{B} ) * \mathbf{I} = (\mathbf{C} ^{\prime } \mathbf{C} ) * \mathbf{I} = \mathbf{I} , \end{aligned}$$

where ‘*’ denotes the Hadamard product, i.e., element-wise product of matrices. Therefore, (4) and (5) imply that there exists a permutation matrix **P** such that

8 $$\begin{aligned} \mathbf{A }_{\mathrm {T}} = \mathbf{AP }, \, \mathbf{B }_{\mathrm {T}} = \mathbf{BP} , \, \mathbf{C }_{\mathrm {T}} = \mathbf{CP }. \end{aligned}$$

It is important to note that parameter estimates are not scale invariant. A rescaling of the data does not imply only an analogous rescaling of the estimates. It follows that there exists a way to rescale the data better, or more appropriate, than another, making crucial the choice. This is witnessed by a remarkably large number of papers devoted to the preprocessing step. See, for instance, Harshman & Lundy (1984), Bro & Smilde (2003), Kiers (2006) and references therein. The goal of preprocessing is similar to that for standard PCA. Namely, data are standardized in order to eliminate unwanted differences among the variables. Difficulties arise because it is no longer obvious what is the best strategy to adopt. For instance, a three-way three-mode array can be normalized within one of the three modes or even within a combination of two modes. Different ways of preprocessing the data lead to different solutions, and a wrong preprocessing may lead to unfeasible results (see, e.g., Kiers, 2006). Obviously, a scale-invariant model would be simpler to apply in practice especially for non-expert users because such a crucial decision on the best preprocessing strategy would be no longer necessary.

For a thorough discussion on three-way three-mode analysis according to a component-based approach, see the monographies of Bro (1998), Smilde, Bro, & Geladi (2004) and Kroonenberg (2008) and the reviews of Acar & Yener (2009), Kolda & Bader (2009), Mørup (2011) and Giordani & Kiers (2018).

## The Structural Approach

### Models

Starting from the original formulations of the models, we can derive what is the covariance structure corresponding to each model. Let us consider the Tucker3 model in (1) by limiting our attention to the *i*th row of $\mathbf{X }_{\mathrm {A}}$, say $\mathbf{x }_{Ai}^{\prime }$, pertaining to the *i*th statistical unit. $\mathbf{x }_{Ai}^{\prime }$ is the vector of length *JK* containing the scores of statistical unit *i* on the *J* manifest variables during the *K* occasions. We get

9 $$\begin{aligned} \mathbf{x }_{Ai}^{\prime } = \mathbf{a }_{i}^{\prime } \mathbf{G }_{\mathrm {A}} (\mathbf{C} \otimes \mathbf{B} )^{\prime } + \mathbf{e }_{Ai}^{\prime }, \end{aligned}$$

where with obvious notation, $\mathbf{e }_{Ai}^{\prime }$ represents the *i*th row of $\mathbf{E }_{\mathrm {A}}$ and $\mathbf{a }_{i}^{\prime }$ is the *i*th row of **A **containing the component scores for statistical unit *i*. To simplify the notation, we omit the subscripts ‘A’ and ‘*i*,’ and we rewrite (9) in terms of column vectors, explicitly considering a vector of intercepts

10 $$\begin{aligned} \mathbf{x} = {\varvec{\upmu }} + (\mathbf{C} \otimes \mathbf{B} )\mathbf{G} ^{\prime } \mathbf{a} + \mathbf{e} . \end{aligned}$$

As usual in standard FA, we assume that the common factors **a** and the specific factors **e** are random with ${E}(\mathbf{a} ) = \mathbf{0}$ and ${E}(\mathbf{e} ) = \mathbf{0}$, without loss of generality because of ${\varvec{\upmu }}$, and ${E}(\mathbf{ae }^{\prime }) = \mathbf{0}$. If ${E}(\mathbf{aa }^{\prime }) = {\varvec{\Phi }}$ and ${E}(\mathbf{ee } ^{\prime }) = {\varvec{\Psi }}$, then the covariance matrix of **x** is given by

11 $$\begin{aligned} {\varvec{\Sigma }} = {E}[(\mathbf{x} - {\varvec{\upmu }}) (\mathbf{x} - {\varvec{\upmu }})^{\prime }] = (\mathbf{C} \otimes \mathbf{B} )\mathbf{G} ^{\prime } {\varvec{\Phi }} \mathbf{G} (\mathbf{C} \otimes \mathbf{B} )^{\prime } + {\varvec{\Psi }}. \end{aligned}$$

In what follows, ${\varvec{\Phi }}$ and ${\varvec{\Psi }}$ will be assumed to be positive definite. The generic element of the matrix ${\varvec{\Sigma }}$ (of order ${\textit{JK}} \times {\textit{JK}}$), $\mathrm{{\varvec{\sigma }}}_{jk,j^{\prime } k^{\prime }}$, holds the covariance between manifest variable *j* at occasion *k* and manifest variable $j^{\prime }$ at occasion $k^{\prime }$ ($j, j^{\prime } = 1, \ldots , J$; $k, k^{\prime } = 1, \ldots , K$). Bearing in mind the standard FA model, it should be clear that the Tucker3 model is a constrained version of standard FA. If we set ${\varvec{\Lambda }} = (\mathbf{C} \otimes \mathbf{B} ) \mathbf{G }^{\prime }$, then (11) can be rewritten as

12 $$\begin{aligned} {\varvec{\Sigma }} = {\varvec{\Lambda \Phi \Lambda }}^{\prime } + {\varvec{\Psi }}, \end{aligned}$$

which is the oblique FA model, where ${\varvec{\Lambda }} = (\mathbf{C} \otimes \mathbf{B} ) \mathbf{G }^{\prime }$ is the matrix of factor loadings having a particular form taking into account the three-way three-mode structure of the data.

Several papers available in the literature investigate the model in (11). Interested readers may refer to Bloxom (1968), Bentler & Lee (1978), Bentler & Lee (1979), Lee & Fong (1983), Bloxom (1984), Bentler, Poon, & Lee (1988), Kroonenberg & Oort (2003) and references therein. Other multilinear decompositions with Kronecker structured covariance matrices are presented and investigated by, for instance, Gerard & Hoff (2015) and Hoff (2016).

The Parafac model can be derived from (10) setting $\mathbf{G} = \mathbf{G }_{\mathrm {A}} = \mathbf{I }_{\mathrm {A}}$ as in (3). We have

13 $$\begin{aligned} \mathbf{x} = {\varvec{\upmu }} + (\mathbf{C} \bullet \mathbf{B} )\mathbf{a} + \mathbf{e} . \end{aligned}$$

This shows that even the Parafac model is the same as the classical FA model, where the observed variables are expressed as a linear combination of a limited number of common factors (**a**) and specific factors (**e**). In order to take into account the three-way three-mode structure of the data, the loadings are constrained to be equal to $\mathbf{C} \bullet \mathbf{B}$. Similarly, the structural version of Parafac can be derived from (11) imposing the restriction $\mathbf{G} = \mathbf{G }_{\mathrm {A}} = \mathbf{I }_{\mathrm {A}}$ obtaining

14 $$\begin{aligned} {\varvec{\Sigma }} = E[(\mathbf{x} - {\varvec{\upmu }}) (\mathbf{x} - {\varvec{\upmu }})^{\prime }] = (\mathbf{C} \bullet \mathbf{B} ) {\varvec{\Phi }} (\mathbf{C} \bullet \mathbf{B} )^{\prime } + {\varvec{\Psi }}. \end{aligned}$$

Comparing (10) with (13), we see that the restriction $\mathbf{G} = \mathbf{G }_{\mathrm {A}} = \mathbf{I }_{\mathrm {A}}$ greatly simplifies model interpretation. This aspect can be highlighted by focusing on a single occasion, say *k*. Let $\mathbf{x }_{k}$ be the vector of the *J* manifest variables measured at occasion *k*, with obvious notation, the Tucker3 and Parafac assume the form

15 $$\begin{aligned} \mathbf{x }_{k}= & {} {\varvec{\upmu }}_{k} + \mathbf{B} \left( {\sum }_{r} \, c_{kr} \mathbf{G }_{r}\right) ^{\prime } \mathbf{a} + \mathbf{e }_{k}, \end{aligned}$$

16 $$\begin{aligned} \mathbf{x }_{k}= & {} {\varvec{\upmu }}_{k} + \mathbf{B} \hbox {diag}(\mathbf{c }_{k}) \mathbf{a} + \mathbf{e }_{k}, \end{aligned}$$

respectively, where diag(**z**) is the diagonal matrix with the elements of **z** on the main diagonal. Under the Tucker3 model, **B** may be interpreted as the matrix of factor loadings for the variables on the common factors $({\varvec{\Sigma }}_{r} \, c_{kr} \mathbf{G }_{r})^{\prime } \mathbf{a}$ varying the covariance structure across the occasions. Even under the Parafac, the factor loadings for the variables are the same (**B**) in each occasion, but the common factors $\hbox {diag} (\mathbf{c }_{k}) \mathbf{a}$ vary only in variance. By exploiting the symmetry of the models with respect to the modes, we may interpret **C** as the factor loading matrix for the occasions.

In the following section, we reconsider the structural Parafac model by analyzing whether the constraints ${\varvec{\Lambda }} = \mathbf{C} \bullet \mathbf{B}$ affect the parameter identifiability under different covariance structure of the specific factors.

### Scale Invariance

The practical applicability of FA is favored by the property of scale invariance. As is well known, the factorial structure of the standard FA model is not destroyed by variable rescaling. For example, if **x** is rescaled as $\mathbf{x }_{\mathrm {L}} = \mathbf{Lx}$ where **L** is a diagonal matrix, then the covariance matrix of $\mathbf{x }_{\mathrm {L}}$, ${\varvec{\Sigma }}_{\mathrm {L}}$, is equal to

17 $$\begin{aligned} {\varvec{\Sigma }}_{\mathrm {L}} = V(\mathbf{Lx }) = \mathbf{L} \hbox {V}(\mathbf{x} )\mathbf{L} = \mathbf{L} ({\varvec{\Lambda \Phi \Lambda }}^{\prime } + {\varvec{\Psi }}) \mathbf{L} = (\mathbf{L} {\varvec{\Lambda }}) {\varvec{\Phi }} (\mathbf{L} {\varvec{\Lambda }})^{\prime } + \mathbf{L} {\varvec{\Psi }} \mathbf{L} = {\varvec{\Lambda }}_{\mathrm {L}} {\varvec{\Phi \Lambda }}_{\mathrm {L}}^{\prime } + {\varvec{\Psi }}_{\mathrm {L}},\nonumber \\ \end{aligned}$$

with ${\varvec{\Lambda }}_{\mathrm {L}} = \mathbf{L} {\varvec{\Lambda }}$ and ${\varvec{\Psi }}_{\mathrm {L}} = \mathbf{L} {\varvec{\Psi }} \mathbf{L}$. Hence, the factor loadings are the same but expressed in the new units of measurements. This guarantees that when the model is estimated by a scale-invariant method, like maximum likelihood, a rescaling of the data implies an analogous rescaling of the estimates. If **L** contains standard deviations, ${\varvec{\Sigma }}_{\mathrm {L}}$ is the correlation matrix of **x** and standard FA applied to covariance or correlation matrices leads to the same conclusions. This is not true for PCA. The components extracted by using ${\varvec{\Sigma }}_{\mathrm {L}}$ or ${\varvec{\Sigma }}$ differ, and therefore, a crucial question to answer before applying PCA is how to preprocess the data.

In the structural case, the Parafac model introduced in (13), and the resulting covariance matrix given in (14), is generally not scale invariant in the sense given by Browne (1982). In fact, if **x** in (13) is rescaled as $\mathbf{x }_{\mathrm {L}} = \mathbf{Lx }$, then

18 $$\begin{aligned} {\varvec{\Sigma }}_{\mathrm {L}} = \mathbf{L} [(\mathbf{C} \bullet \mathbf{B} ) {\varvec{\Phi }} (\mathbf{C} \bullet \mathbf{B} )^{\prime } + {\varvec{\Psi }} ]\mathbf{L} = [\mathbf{L} (\mathbf{C} \bullet \mathbf{B} )] {\varvec{\Phi }} [\mathbf{L} (\mathbf{C} \bullet \mathbf{B} )]^{\prime } + {\varvec{\Psi }}_{\mathrm {L}}; \end{aligned}$$

it follows that, in general, the structural Parafac model in (14) is not scale invariant because the equation

19 $$\begin{aligned} \mathbf{L} (\mathbf{C} \bullet \mathbf{B} ) = \mathbf{C }_{\mathrm {L}} \bullet \mathbf{B }_{\mathrm {L}} \end{aligned}$$

does not always have a solution. In other terms, the matrix **L** usually destroys the Khatri–Rao structure of the factor loadings unless **L** has a particular structure.

Bearing in mind that the same variables are collected at different occasions, it may be reasonable to perform the same scaling of the variables in the various occasions and the same scaling of the occasions across the different variables. This is equivalent to say that

20 $$\begin{aligned} \mathbf{L} = (\mathbf{L }_{\mathrm {O}}\otimes \mathbf{L }_{\mathrm {V}}), \end{aligned}$$

where $\mathbf{L }_{\mathrm {V}} = \hbox {diag} ([l_{\mathrm {V1}}, \ldots , l_{{\mathrm {V}} j}, \ldots , l_{{\mathrm {V}} J}]^{\prime })$ and $\mathbf{L }_{\mathrm {O}} = \hbox {diag} ([l_{\mathrm {O1}}, \ldots , l_{{\mathrm {O}} k}, \ldots , l_{{\mathrm {O}} K}]^{\prime })$. The generic element $l_{{\mathrm {V}} j}$ gives the scaling of variable $j \, (j = 1, \ldots , J)$. Similarly, $l_{{\mathrm {O}} k}$ represents the scaling of occasion $k \, (k = 1, \ldots , K)$. It follows that $x_{jk}$, i.e., variable *j* at occasion *k*, is rescaled as $l_{{\mathrm {V}} j} l_{{\mathrm {O}} k}x_{jk}$. In this particular case, by substituting (20) into (19), we get

21 $$\begin{aligned} \mathbf{L} (\mathbf{C} \bullet \mathbf{B} ) = (\mathbf{L }_{\mathrm {O}}\otimes \mathbf{L }_{\mathrm {V}})(\mathbf{C} \bullet \mathbf{B} ) = (\mathbf{L }_{\mathrm {O}}{} \mathbf{C} \bullet \mathbf{L }_{\mathrm {V}}{} \mathbf{B} ) = \mathbf{C }_{\mathrm {L}}\bullet \mathbf{B }_{\mathrm {L}}, \end{aligned}$$

with $\mathbf{B }_{\mathrm {L}} = \mathbf{L }_{\mathrm {V}} \mathbf{B}$ and $\mathbf{C }_{\mathrm {L}} = \mathbf{L }_{\mathrm {O}} \mathbf{C}$. Therefore, the scaling is absorbed by the Khatri–Rao product and the particular factor loading structure is not destroyed.

However, in general, the structural Parafac model is not scale invariant and a researcher may be interested in knowing how to modify its structure to derive a scale-invariant version. Following Lee & Fong (1983), the Parafac formulation in (13) can be modified by incorporating a positive definite diagonal matrix **D** in the model able to absorb scale changes. We get

22 $$\begin{aligned} \mathbf{x} = {\varvec{\upmu }} + \mathbf{D} [(\mathbf{C} \bullet \mathbf{B} )\mathbf{a} + \mathbf{e} ]. \end{aligned}$$

From (22), the covariance matrix of **x** is

23 $$\begin{aligned} {\varvec{\Sigma }} = \mathbf{D} [(\mathbf{C} \bullet \mathbf{B} ) {\varvec{\Phi }} (\mathbf{C} \bullet \mathbf{B} )^{\prime } + {\varvec{\Psi }}]\mathbf{D} . \end{aligned}$$

In this way, the Parafac model becomes scale invariant. In fact, when **x** is rescaled as $\mathbf{x }_{\mathrm {L}} = \mathbf{Lx }$, (22) becomes

24 $$\begin{aligned} \mathbf{x }_{\mathrm {L}} = \mathbf{Lx} = \mathbf{L} \{ {\varvec{\upmu }} + \mathbf{D} [(\mathbf{C} \bullet \mathbf{B} )\mathbf{a} + \mathbf{e} ]\} = {\varvec{\upmu }}_{\mathrm {L}} + \mathbf{D }_{\mathrm {L}} [(\mathbf{C} \bullet \mathbf{B} )\mathbf{a} + \mathbf{e} ], \end{aligned}$$

with ${\varvec{\upmu }}_{\mathrm {L}} = \mathbf{L} {\varvec{\upmu }}$ and $\mathbf{D }_{\mathrm {L}} = \mathbf{LD }$. The covariance matrix of $\mathbf{x }_{\mathrm {L}}$ is

25 $$\begin{aligned} {\varvec{\Sigma }}_{\mathrm {L}} = \mathbf{D }_{\mathrm {L}} [(\mathbf{C} \bullet \mathbf{B} ) {\varvec{\Phi }} (\mathbf{C} \bullet \mathbf{B} )^{\prime } + {\varvec{\Psi }}] \mathbf{D }_{\mathrm {L}}. \end{aligned}$$

Hence, the model is not affected by **L** because the scaling is absorbed in the diagonal matrix $\mathbf{D }_{\mathrm {L}}$. It is important to note that if we impose a particular structure on **D**, then the same should be on $\mathbf{D }_{\mathrm {L}} = \mathbf{LD }$ for every possible **L**. It follows that we cannot impose any particular structure on **D**. In order to identify the diagonal matrix **D**, either the constraint $((\mathbf{C} \bullet \mathbf{B} ) {\varvec{\Phi }} (\mathbf{C} \bullet \mathbf{B} )^{\prime } + {\varvec{\Psi }})* \mathbf{I} = \mathbf{I}$ (Lee & Fong, 1983) or ${\varvec{\Psi }} = \mathbf{I}$ (Bentler, Poon, & Lee, 1988) is imposed. The two formulations are not equivalent. The former allows us to interpret the model as a reparameterization of the correlation matrix, and the latter has an interpretation less clear but avoids complex constraints on the estimates of the parameter matrices except for ${\varvec{\Psi }}$.

## Factor Uniqueness of the Structural Parafac Model

A fundamental property of the Parafac model is its factor uniqueness. In this section, we start showing that Parafac maintains this property even in the structural formulation when, as in the standard FA model in (12), the specific factors are assumed to be uncorrelated, i.e., the matrix ${\varvec{\Psi }}$ is diagonal. However, in three-way three-mode FA, we note that the uncorrelation of the specific factors might be a too restrictive assumption. For instance, it might be reasonable to assume that ${\varvec{\Psi }}$ is block diagonal, i.e., the specific factors of the different variables are correlated within the same occasion

26 $$\begin{aligned} {\varvec{\Psi }} = \hbox {blockdiag} ({\varvec{\Psi }}_{11}, \ldots , {\varvec{\Psi }}_{kk}, \ldots , {\varvec{\Psi }}_{K\! K}), \end{aligned}$$

where $\hbox {blockdiag} (\mathbf{Z }_{1}, \ldots , \mathbf{Z }_{K})$ denotes the block-diagonal matrix with diagonal elements equal to the square matrices $\mathbf{Z }_{1}, \ldots , \mathbf{Z }_{K}$ and ${\varvec{\Psi }}_{kk}$ denotes the covariance matrix of order ($J \times J$) for the specific factors at occasion *k*, $k = 1, \ldots , K$. The Parafac covariance model in (14) with the correlation structure of the specific factors given in (26) represents a more realistic model able to fit reasonably well in many practical situations.

To interpret/understand correlations between specific factors, we note that they are allowed only between specific factors in the same occasion. In other words, the idea is that correlations between variables measured in different occasions are due only to common factors, while correlations between variables in the same occasion can also be due to other factors (i.e., the specific factors in that occasion) that are not present in other occasions and therefore out of our interest. An accurate estimation of the ‘true’/interesting common factors should consider explicitly the presence of such factors that are in ‘common’ only with the variables measured in the same occasion. From a statistical point of view, such covariances are only nuisance parameters. In the literature, the assumption of correlated specific factors is not new. See, e.g., Browne (1984a) and Kroonenberg & Oort (2003).

In the following, we investigate under what conditions factor uniqueness holds even with a block-diagonal structure for ${\varvec{\Psi }}$. Finally, we address the scale invariance problem studying the factor uniqueness property of the scale-invariant version of Parafac in (22–23).

It is important to note that what follows can be extended to the case where the specific factors of the same variable are correlated across the different occasions. Such an extension can be easily obtained by exploiting the symmetry of the model with respect to variables and occasions.

### ${\varvec{\Psi }}$ Diagonal

Let us start by showing the factor uniqueness property of the structural Parafac when ${\varvec{\Psi }}$ is diagonal. Note that disjoint submatrices are submatrices having no entry in common.

#### Result 1

Suppose that the following hold:

- 27 $$\begin{aligned} \qquad (\mathbf{C} \bullet \mathbf{B} ) {\varvec{\Phi }} (\mathbf{C} \bullet \mathbf{B} )^{\prime } + {\varvec{\Psi }} = (\mathbf{C }_{\mathrm {T}} \bullet \mathbf{B }_{\mathrm {T}}) \, {\varvec{\Phi }}_{\mathrm {T}} (\mathbf{C }_{\mathrm {T}} \bullet \mathbf{B }_{\mathrm {T}})^{\prime } + {\varvec{\Psi }}_{\mathrm {T}}, \end{aligned}$$
- 28 $$\begin{aligned} \qquad k \hbox {-rank}(\mathbf{B} ) + k\hbox {-rank}(\mathbf{C} ) \ge S+2, \end{aligned}$$
-       if any row of $\mathbf{C} \bullet \mathbf{B}$ is deleted, there remain two disjoint submatrices of rank *S*,

where ${\varvec{\Psi }}$ and ${\varvec{\Psi }}_{\mathrm {T}}$ are diagonal matrices and all the remaining ones have *S* columns.

Under the scaling set in (7), there exists a permutation matrix **P** such that

29 $$\begin{aligned} \mathbf{B }_{\mathrm {T}} = \mathbf{BP }, \mathbf{C }_{\mathrm {T}} = \mathbf{CP} , {\varvec{\Phi }}_{\mathrm {T}} = \mathbf{P} {\varvec{\Phi }} \mathbf{P} . \end{aligned}$$

#### Proof

Let us rewrite equality (a) in terms of standard FA models with uncorrelated common factors

30 $$\begin{aligned} {\varvec{\Sigma }} = \big [(\mathbf{C} \bullet \mathbf{B} ) {\varvec{\Phi }}^{1/2}\big ] \big [(\mathbf{C} \bullet \mathbf{B} ) {\varvec{\Phi }}^{1/2}\big ]^{\prime } + {\varvec{\Psi }} = \big [(\mathbf{C }_{\mathrm {T}} \bullet \mathbf{B }_{\mathrm {T}}) {\varvec{\Phi }}_{\mathrm {T}}^{1/2}\big ] \big [(\mathbf{C }_{\mathrm {T}}\bullet \mathbf{B }_{\mathrm {T}}) {\varvec{\Phi }}_{\mathrm {T}}^{1/2}\big ]^{\prime } + {\varvec{\Psi }}_{\mathrm {T}}. \end{aligned}$$

Condition (c) implies that if any row of $(\mathbf{C} \bullet \mathbf{B} ) \, {\varvec{\Phi }}^{1/2}$ is deleted, there remain two disjoint submatrices of rank *S* since $\hbox {rank} ({\varvec{\Phi }}) = \hbox {rank} ({\varvec{\Phi }}^{1/2}) = S$. From Theorem 5.1 of Anderson & Rubin (1956), we know that when this is true, there exists a column-wise orthonormal matrix **T** such that

31 $$\begin{aligned} (\mathbf{C} \bullet \mathbf{B} ) {\varvec{\Phi }}^{1/2} = (\mathbf{C }_{\mathrm {T}} \bullet \mathbf{B }_{\mathrm {T}}) {\varvec{\Phi }} _{\mathrm {T}}^{1/2} \mathbf{T} . \end{aligned}$$

Inequality (b) implies

32 $$\begin{aligned} k\hbox {-rank} \big ({\varvec{\Phi }}^{1/2}\big ) + k\hbox {-rank} (\mathbf{B} ) + k\hbox {-rank} (\mathbf{C} ) \ge 2 S+2, \end{aligned}$$

indicating that Kruskal’s condition is met and the two decompositions in (31) are equal up to a column permutation under constraints (7). Let us indicate with **P** such a permutation matrix, we have

33 $$\begin{aligned} \mathbf{B }_{\mathrm {T}} = \mathbf{BP} , \mathbf{C }_{\mathrm {T}} = \mathbf{CP} , {\varvec{\Phi }}_{\mathrm {T}}^{1/2} = \mathbf{T} ^{\prime } {\varvec{\Phi }}^{1/2}{} \mathbf{P} =\>{\varvec{\Phi }}_{\mathrm {T}} = \mathbf{P} ^{\prime } {\varvec{\Phi }} \mathbf{P} . \end{aligned}$$

$$\square $$

Finally, we note that, in the previous proposition, Kruskal’s condition can be replaced with any other sufficient condition available in the literature.

### ${\varvec{\Psi }}$ Block Diagonal

When ${\varvec{\Psi }}$ is block diagonal, the conditions of Anderson & Rubin (1956) cannot be longer applied as it assumes a diagonal structure for ${\varvec{\Psi }}$. To prove the factor uniqueness, the following proposition is considered.

#### Proposition

(Browne, 1980) Let us consider the decomposition ${\varvec{\Sigma }} = {\varvec{\Lambda \Lambda }}^{\prime } +{\varvec{\Psi }}$, where ${\varvec{\Lambda }}$ is partitioned as ${\varvec{\Lambda }} = [{\varvec{\Lambda }}_{1}^{\prime }, \ldots , {\varvec{\Lambda }}_{k}^{\prime }, \ldots , {\varvec{\Lambda }} _{K}^{\prime }]^{\prime }$ and ${\varvec{\Psi }}$ has the corresponding block-diagonal structure ${\varvec{\Psi }} = \hbox {blockdiag} ({\varvec{\Psi }}_{11}, \ldots , {\varvec{\Psi }}_{k\! k}, \ldots , {\varvec{\Psi }}_{\textit{KK}})$. The identification of ${\varvec{\Psi }}$ and ${\varvec{\Lambda }}$ up to post-multiplication by an orthogonal matrix **T** holds if at least three of the submatrices ${\varvec{\Lambda }}_{k}$ are of full column rank.

The above proposition has been formulated in the context of the FA model for multiple batteries of tests. The matrices ${\varvec{\Lambda }}_{k}$ and ${\varvec{\Psi }}_{kk}$ refer to the *k*th battery. The factor loading matrix is obtained by juxtaposing the factor loading matrices of the various batteries, and the specific factors are uncorrelated between the batteries, but correlated within the batteries. The proposition can be applied in order to prove the factor uniqueness of the structural Parafac model when the specific factors are correlated within the occasions. A sufficient condition for the factor uniqueness of the Parafac solution is given in Result 2.

#### Result 2

Suppose that the following hold:

- 34 $$\begin{aligned} \qquad (\mathbf{C} \bullet \mathbf{B} ) {\varvec{\Phi }} (\mathbf{C} \bullet \mathbf{B} )^{\prime } + {\varvec{\Psi }} = (\mathbf{C }_{\mathrm {T}}\bullet \mathbf{B }_{\mathrm {T}}) {\varvec{\Phi }}_{\mathrm {T}}(\mathbf{C }_{\mathrm {T}}\bullet \mathbf{B }_{\mathrm {T}})^{\prime } + {\varvec{\Psi }}_{\mathrm {T}}, \end{aligned}$$
- 35 $$\begin{aligned} \qquad k\hbox {-rank}(\mathbf{B} ) + k\hbox {-rank}(\mathbf{C} ) \ge S+2, \end{aligned}$$
- 36 $$\begin{aligned} \qquad \hbox {rank} (\mathbf{B} \hbox {diag} (\mathbf{c }_{k})) = S \, \hbox {for at least three occasions}, \end{aligned}$$

where ${\varvec{\Psi }} = \hbox {blockdiag} ({\varvec{\Psi }}_{11}, \ldots , {\varvec{\Psi }}_{kk}, \ldots , {\varvec{\Psi }}_{K\! K})$ is block diagonal with blocks defined by the occasions, ${\varvec{\Psi }}_{\mathrm {T}}$ has the same structure, all the remaining matrices have *S* columns, and $\mathbf{c }_{k}$ is the vector containing the elements of the *k*th row of **C**.

Under the scaling set in (7), there exists a permutation matrix **P** such that

37 $$\begin{aligned} \mathbf{B }_{\mathrm {T}} = \mathbf{BP }, \mathbf{C }_{\mathrm {T}} = \mathbf{CP }, {\varvec{\Phi }}_{\mathrm {T}} = \mathbf{P} {\varvec{\Phi }} \mathbf{P} . \end{aligned}$$

#### Proof

Let us rewrite equality (a) in terms of standard FA models with uncorrelated common factors

38 $$\begin{aligned} {\varvec{\Sigma }} = \big [(\mathbf{C} \bullet \mathbf{B} ) {\varvec{\Phi }}^{1/2}\big ] \big [(\mathbf{C} \bullet \mathbf{B} ) {\varvec{\Phi }}^{1/2}\big ]^{\prime } + {\varvec{\Psi }} = \big [(\mathbf{C }_{\mathrm {T}}\bullet \mathbf{B }_{\mathrm {T}}) {\varvec{\Phi }}_{\mathrm {T}}^{1/2}\big ]\big [(\mathbf{C }_{\mathrm {T}}\bullet \mathbf{B }_{\mathrm {T}}) {\varvec{\Phi }}_{\mathrm {T}}^{1/2}\big ]^{\prime } + {\varvec{\Psi }}_{\mathrm {T}}, \end{aligned}$$

where the matrix of factor loadings $(\mathbf{C} \bullet \mathbf{B} ) \, {\varvec{\Phi }}^{1/2}$, if partitioned according to the blocks of ${\varvec{\Psi }}$, takes the form $(\mathbf{C} \bullet \mathbf{B} ) \, {\varvec{\Phi }}^{1/2}= [(\mathbf{B} \hbox {diag} (\mathbf{c }_{1}) {\varvec{\Phi }}^{1/2})^{\prime }, \ldots , (\mathbf{B} \hbox {diag} (\mathbf{c }_{k}) {\varvec{\Phi }}^{1/2})^{\prime }, \ldots , (\mathbf{B} \hbox {diag} (\mathbf{c }_{K}) {\varvec{\Phi }}^{1/2})^{\prime }]^{\prime }$. Condition (c) implies $\hbox {rank} (\mathbf{B} \hbox {diag} (\mathbf{c }_{k}) {\varvec{\Phi }}^{1/2}) = S$ for at least three occasions since $\hbox {rank} ({\varvec{\Phi }}) = \hbox {rank} ({\varvec{\Phi }}^{1/2}) = S$. This allows us to use Browne’s proposition in order to show that (38) implies the existence of a column-wise orthonormal matrix **T** such that

39 $$\begin{aligned} (\mathbf{C} \bullet \mathbf{B} ) {\varvec{\Phi }}^{1/2} = (\mathbf{C }_{\mathrm {T}}\bullet \mathbf{B }_{\mathrm {T}}) {\varvec{\Phi }}_{\mathrm {T}}^{1/2}{} \mathbf{T} . \end{aligned}$$

Inequality (b) implies

40 $$\begin{aligned} k\hbox {-rank} \big ({\varvec{\Phi }}^{1/2}\big ) + k\hbox {-rank} (\mathbf{B} ) + k\hbox {-rank} (\mathbf{C} ) \ge 2 S+2, \end{aligned}$$

indicating that Kruskal’s condition is met and the two decompositions in (39) are equal up to a column permutation under constraints (7). Let us indicate with **P** such a permutation matrix, we have

41 $$\begin{aligned} \mathbf{B }_{\mathrm {T}} = \mathbf{BP }, \mathbf{C }_{\mathrm {T}} = \mathbf{CP }, {\varvec{\Phi }}_{\mathrm {T}}^{1/2} = \mathbf{T }^{\prime } {\varvec{\Phi }}^{1/2} \mathbf{P} \Rightarrow {\varvec{\Phi }}_{\mathrm {T}} = \mathbf{P }^{\prime } {\varvec{\Phi }} \mathbf{P} . \end{aligned}$$

$$\square $$

### Scale Invariance

It remains to prove that the scale-invariant versions of the structural Parafac model in (25), with constraint (a) $((\mathbf{C} \bullet \mathbf{B} ) {\varvec{\Phi }} (\mathbf{C} \bullet \mathbf{B} )^{\prime } + {\varvec{\Psi }})* \mathbf{I} = \mathbf{I}$ (Lee & Fong, 1983) or (b) ${\varvec{\Psi }} *\mathbf{I} = \mathbf{I}$ (Bentler, Poon, & Lee, 1988), satisfy the factor uniqueness property. Note that the latter constraint is modified with respect to the original version to also handle the case where ${\varvec{\Psi }}$ is block diagonal. The following results hold for either of the two cases where ${\varvec{\Psi }}$ is diagonal or block diagonal.

Let us consider the equality

42 $$\begin{aligned} \mathbf{D} [(\mathbf{C} \bullet \mathbf{B} ) {\varvec{\Phi }} (\mathbf{C} \bullet \mathbf{B} )^{\prime } + {\varvec{\Psi }}]\mathbf{D} = \mathbf{D }_{\mathrm {T}}[(\mathbf{C }_{\mathrm {T}}\bullet \mathbf{B }_{\mathrm {T}}) {\varvec{\Phi }}_{\mathrm {T}}(\mathbf{C }_{\mathrm {T}}\bullet \mathbf{B }_{\mathrm {T}})^{\prime } + {\varvec{\Psi }}_{\mathrm {T}}]\mathbf{D }_{\mathrm {T}} \end{aligned}$$

In order to show that the previous results hold even in this case, it is sufficient to show that the use of either constraint (a) or (b) implies $\mathbf{D} = \mathbf{D }_{\mathrm {T}}$.

In case (a), by focusing the attention on the main diagonal elements of the covariance matrices on the left and right of equality (42), we deduce that

43 $$\begin{aligned} \mathbf{DD } = \mathbf{D }_{\mathrm {T}} \mathbf{D }_{\mathrm {T}} \Rightarrow \mathbf{D} = \mathbf{D }_{\mathrm {T}} \end{aligned}$$

because **D** and $\mathbf{D }_{\mathrm {T}}$ are required to be positive definite.

Case (b) is more complex to show because now the constraint applies only on the covariance matrix of the specific factors and it becomes effective in the identification only if the decomposition of the total covariance is unique. To this end, we rewrite (42) in terms of FA models. Namely, setting ${\varvec{\Lambda }} = \mathbf{D} (\mathbf{C} \bullet \mathbf{B} ) {\varvec{\Phi }} ^{1/2}$ and $\mathbf{Z } = \mathbf{D} {\varvec{\Psi }} \mathbf{D}$, with obvious notation, we get

44 $$\begin{aligned} {\varvec{\Sigma }} = {\varvec{\Lambda \Lambda }}^{\prime } + \mathbf{Z } = {\varvec{\Lambda }}_{\mathrm {T}} {\varvec{\Lambda }}_{\mathrm {T}}^{\prime } + \mathbf{Z }_{\mathrm {T}}. \end{aligned}$$

If the decomposition of the total covariance into variability due to the common factors and variability due to the specific factors is unique, then it must be $\mathbf{Z } = \mathbf{Z }_{\mathrm {T}}$, i.e., $\mathbf{D} {\varvec{\Psi }} \mathbf{D} = \mathbf{D }_{\mathrm {T}} {\varvec{\Psi }}_{\mathrm {T}} \mathbf{D }_{\mathrm {T}}$; hence, $\mathbf{D} = \mathbf{D }_{\mathrm {T}}$ because ${\varvec{\Psi }}*\mathbf{I} = {\varvec{\Psi }}_{\mathrm {T}}*\mathbf{I} = \mathbf{I}$. It remains to discuss under what conditions the total covariance decomposition is unique. In particular, we are going to show that this is obtained when condition (c) in either Result 1 or 2 is satisfied.

In the case of a diagonal ${\varvec{\Psi }}$, when condition (c) of Result 1 is met, if any row of ${\varvec{\Lambda }} = \mathbf{D} (\mathbf{C} \bullet \mathbf{B} ) {\varvec{\Phi }}^{1/2}$ is deleted, there remain two disjoint submatrices of rank *S*. This allows us to apply Theorem 5.1 of Anderson & Rubin (1956) obtaining the required factor uniqueness.

In the case of a block diagonal ${\varvec{\Psi }}$, when condition (c) of Result 2 is met, at least three submatrices of $\mathbf{D} (\mathbf{C} \bullet \mathbf{B} ) \, {\varvec{\Phi }}^{1/2} = [(\mathbf{D }_{1} \mathbf{B} \hbox {diag} (\mathbf{c }_{1}) \, {\varvec{\Phi }}^{1/2})^{\prime }, \ldots , (\mathbf{D }_{k} \mathbf{B} \hbox {diag} (\mathbf{c }_{k}) \, {\varvec{\Phi }}^{1/2})^{\prime }, \ldots , (\mathbf{D }_{K} \mathbf{B} \hbox {diag} (\mathbf{c }_{K}) \, {\varvec{\Phi }}^{1/2})^{\prime }]^{\prime }$, where $\mathbf{D} = \hbox {blockdiag} (\mathbf{D }_{1}, \ldots , \mathbf{D }_{k}, \ldots , \mathbf{D }_{K})$, are of full column rank. This allows us to apply Browne’s proposition obtaining the required factor uniqueness.

## Estimation of the Structural Parafac Model

The parameter estimation of the structural Parafac model can be done in several ways. Ordinary least squares (OLS), generalized least squares (GLS), maximum likelihood (ML) approaches can be applied. They differ in the discrepancy function to be minimized between the sample covariance matrix and the one induced by the Parafac model. If observations are independent and identically distributed as a multinormal distribution, the estimate is obtained in such a way to minimize

45 $$\begin{aligned} {f} ({\uptheta }) = \hbox {tr} \{[\mathbf{S} {-} {\varvec{\Sigma }} ({\uptheta })]\mathbf{W }^{-1}\}^{2}/2 \end{aligned}$$

where ${\uptheta } = \{\mathbf{B }, \mathbf{C }, {\varvec{\Phi }}, {\varvec{\Psi }}\}$ denotes the set of parameters, **S** is the sample covariance matrix of order (${\textit{JK}} \times {\textit{JK}}$), ${\varvec{\Sigma }} ({\uptheta })$ is the estimated covariance matrix according to the Parafac model, and **W** is a weight matrix. If $\mathbf{W} = \mathbf{I}$ in (45), then the OLS estimate is found. The OLS approach is, however, not recommended because it is not scale invariant regardless the model properties. If we set $\mathbf{W} = \mathbf{S}$, then the so-called best GLS estimate is found, while the ML estimate is almost certainly obtained setting $\mathbf{W} = {\varvec{\Sigma }} ({\uptheta })$ when *I* is large enough (Browne, 1974). The ‘best’ GLS and ML estimators are asymptotically equivalent and therefore share the same asymptotic properties. The most relevant difference is that the GLS approach does not exactly reproduce the sample variances of the manifest variables (see, e.g., Bentler & Lee, 1979). If the multivariate normality assumption is violated, the asymptotically distribution-free (ADF) estimation method (Browne, 1984b) can be adopted. The ADF method minimizes the discrepancy function in (45) with a particular choice of **W** involving the sample fourth-order moments about the mean (see, e.g., Lee, 2007, p. 45). A Bayesian strategy can also be adopted (see, e.g., Hoff, 2016).

A closed-form solution to the minimization of (45) does not exist. However, Bentler, Poon, & Lee (1988) show that well-known software packages designed for structural equation models (for a review, see Narayanan, 2012) can be adapted for solving three-way three-mode FA estimation problems such as the Parafac one in (45).

## Related Models

The structural Parafac model is estimated by using a scale-invariant method of estimation, i.e., GLS or ML, and it is not the only attempt to go beyond the component Parafac and its OLS estimation. In the component approach, Bro, Sidiropoulos, & Smilde (2002) address the estimation problem of various models for which OLS fitting procedures exist, such as the Parafac one, when errors are no longer homoscedastic and uncorrelated. They minimize a discrepancy function like

46 $$\begin{aligned} {g}({\uptheta }) = \hbox {tr} \{[\mathbf{X }_{\mathrm {A}} {-} \mathbf{Y }_{\mathrm {A}}({\uptheta })]\mathbf{W }^{-1}\} ^{2} \end{aligned}$$

where ${\uptheta } = \{ \mathbf{A }, \mathbf{B} , \mathbf{C} \}$ and $\mathbf{Y }_{\mathrm {A}}({\uptheta } ) = \mathbf{A} (\mathbf{C} \bullet \mathbf{B} )^{\prime }$. Following the same framework, Vega-Montoto & Wentzell (2003) propose some ALS algorithms for estimating the parameters of the component Parafac model assuming different error structures. Further theoretical and practical results can also be found in two companion papers (Vega-Montoto, Gu, & Wentzell, 2005; Vega-Montoto & Wentzell, 2006).

In between the component and structural approaches, we found the proposal of Harshman (1972), named Parafac2, modeling the within-occasion covariance matrices as, with obvious notation,

47 $$\begin{aligned} \mathbf{S }_{k}= & {} {\varvec{\Sigma }}_{k}({\uptheta } ) + \mathbf{E }_{k}, \end{aligned}$$

48 $$\begin{aligned} {\varvec{\Sigma }}_{k}({\uptheta } )= & {} \mathbf{B} \hbox {diag}(\mathbf{c }_{k}) {\varvec{\Phi }} \hbox {diag} (\mathbf{c }_{k}) \mathbf{B }^{\prime }, \end{aligned}$$

where $\mathbf{S }_{k}$ is the sample covariance matrix of the manifest variables at occasion *k* and $\mathbf{E }_{k}$ is an error term. The model is estimated by OLS, i.e., by minimizing the loss

49 $$\begin{aligned} {h} ({\uptheta }) = {\sum }_{k} \hbox {tr} \{[\mathbf{S }_{k} {-} {\varvec{\Sigma }}_{k}({\uptheta })] \mathbf{W }^{-1}\}^{2}, \end{aligned}$$

with $\mathbf{W} = \mathbf{I}$. It is important to note that (48) differs from (14) because in the former, covariances among different occasions and among specific factors are not considered. Mayekawa (1987) extends the Parafac2 model by introducing the variances of the specific factors and providing the ML estimation.

About the structural approach, it is worth mentioning the work of Stegeman & Lam (2014), where an estimation procedure, based on minimum rank FA, for the structural Parafac model in (14) is proposed under the assumption of uncorrelated specific factors. In the proposal, properties of the estimators are not studied and their standard errors are not derived. Moreover, the adequacy of the model is not evaluated by goodness-of-fit tests.

## Application

The type (b) scale-invariant structural Parafac model is applied to the data introduced by Bentler & McClain (1976). The data refer to the correlation matrix observed on a sample of $I = 68$ fifth-grade children with respect to $J = 4$ personality variables (*E*: Extraversion; *A*: Test anxiety; *I*: Impulsivity; *M*: Academic achievement motivation) measured by Peer report (*P*), Teacher rating (*T*) and Self-rating (*S*), hence $K = 3$. They are reported in Table 1. The data in Table 1 represent an example of multitrait multimethod matrix. The traits are the personality variables, and the methods refer to the judge ratings.

**Table 1:** Bentler & McClain (1976) correlation data.

	Peer report (*P*)				Teacher rating (*T*)				Self-rating (*S*)			
	*E*	*A*	*I*	*M*	*E*	*A*	*I*	*M*	*E*	*A*	*I*	*M*
*P*												
*E*	1											
*A*	$-$ 0.38	1										
*I*	0.42	$-$ 0.21	1									
*M*	$-$ 0.25	0.54	$-$ 0.54	1								
*T*												
*E*	0.64	$-$ 0.15	0.26	$-$ 0.05	1							
*A*	$-$ 0.29	0.66	$-$ 0.19	0.44	$-$ 0.25	1						
*I*	0.38	$-$ 0.09	0.56	$-$ 0.19	0.59	$-$ 0.14	1					
*M*	$-$ 0.22	0.51	$-$ 0.33	0.66	0.06	0.62	$-$ 0.05	1				
*S*												
*E*	0.45	$-$ 0.05	0.12	0.10	0.50	$-$ 0.05	0.36	0.17	1			
*A*	0.04	0.38	$-$ 0.03	0.14	0.08	0.30	0.09	0.16	0.02	1		
*I*	0.33	$-$ 0.13	0.35	$-$ 0.18	0.41	$-$ 0.14	0.45	$-$ 0.13	0.43	0.16	1	
*M*	$-$ 0.21	0.37	$-$ 0.44	0.58	$-$ 0.01	0.41	$-$ 0.10	0.62	0.06	0.04	$-$ 0.37	1

Several scale-invariant structural Parafac models are estimated from the correlation data in Table 1. They are obtained by varying the number of factors (either $S = 2$ or $S = 3$), the covariance structure of the common factors (either orthogonal or oblique) and the covariance matrix of the specific factors (i) diagonal, the specific factors are uncorrelated; (ii) block diagonal, the specific factors are correlated only within the methods; (iii) banded, the specific factors are correlated only within the traits). The scale invariance is obtained by adding the matrix **D** as in (23) and the constraint ${\varvec{\Psi }} *\mathbf{I} = \mathbf{I}$ for identification purposes. Estimation is addressed by ML assuming that the vectors $\mathbf{x }_{i}, i = 1, \ldots , I$, are independent and identically distributed as a multivariate normal. The analysis is performed by using the procedure CALIS for structural equation models available in the SAS software. After some experimentations, we found that the best strategy to limit the risk of non-convergence or convergence to a local optimum is to adopt the Newton–Raphson algorithm and operate as follows. For each model with ${\varvec{\Psi }}$ diagonal, the best-fitting solution is determined by considering 100 runs of the Newton–Raphson algorithm. For every run, the starting point is the OLS solution, i.e., the minimizer of (45) with $\mathbf{W} = \mathbf{I}$, randomly initialized. This choice limits the risk of attaining local optima. When $S = 2, 82$ (orthogonal common factors) and 83 (oblique common factors) times (out of 100), the global optimum is found, whilst when $S = 3$, such numbers decrease to 60 and 46, respectively. Such calculations are made by considering the first four decimal digits. The solution corresponding to the minimum loss function value is considered the best-fitting solution. Such best-fitting solutions are then used as starting points of the corresponding models with ${\varvec{\Psi }}$ block diagonal or banded. We observed that alternative starting points led to local optima.

In the scientific community, there is no agreement on what is the best way to select a model. The suggestion is to use several different methods and choose the model on which there is the maximum agreement. Following this, we consider the $\chi ^{2}$ test of overall fit (Browne, 1982), AIC (Akaike, 1974) and BIC (Schwarz, 1978), which are widely used for model selection in this domain. See, for instance, Akaike (1987), Bartholomew, Knott, & Moustaki (2011) and, in the three-way three-mode context, Kroonenberg & Oort (2003). The $\chi ^{2}$ test refers to the null hypothesis

$$\begin{aligned} {{H}}_{0}: \, {\varvec{\Sigma }} = {\varvec{\Sigma }} ({\uptheta }), \end{aligned}$$

against the general alternative

${{H}}_{1}$: ${\varvec{\Sigma }}$ is any positive definite matrix different from ${\varvec{\Sigma }} ({\uptheta } )$.

**Table 2:** Fit, AIC, BIC and number of parameters for different Parafac models applied to the Bentler & McClain (1976) correlation data.

Number of factors	Structure of common factors	Covariance matrix of specific factors	$\chi ^{2}$ (*p* value)	AIC	BIC	Number of parameters
$S = 2$	Orthogonal	Diagonal	1.85 ($< 0.0001$)	171.78	225.05	24
$S = 2$	Oblique	Diagonal	1.81 ($< 0.0001$)	171.11	226.60	25
$S = 2$	Orthogonal	Banded	1.07 (0.0031)	143.43	223.33	36
$S = 2$	Oblique	Banded	1.04 (0.0034)	143.71	225.83	37
$S = 2$	Orthogonal	Block diag.	1.12 (0.0001)	158.94	252.16	42
$S = 2$	Oblique	Block diag.	1.09 (0.0002)	159.04	254.48	43
$S = 3$	Orthogonal	Diagonal	1.12 (0.0080)	134.76	**201**.**34**	30
$S = 3$	Oblique	Diagonal	1.06 (0.0079)	137.05	210.29	33
$S = 3$	Orthogonal	Banded	**0.74 (0.0639)**	**133**.**70**	226.92	42
$S = 3$	Oblique	Banded	**0.67 (0.0766)**	135.20	235.08	45
$S = 3$	Orthogonal	Block diag.	**0.64 (0.0625)**	138.68	245.22	48
$S = 3$	Oblique	Block diag.	**0.58 (0.0615)**	141.15	254.34	51

The summary of the twelve models considered in terms of *p*-value of the $\chi ^{2}$ test, AIC, BIC and number of parameters is reported in Table 2. By inspecting Table 2, we conclude that there is an agreement toward the use of $S = 3$ factors. In particular, at the level $\alpha = 0.05$, the only four models for which the null hypothesis of the $\chi ^{2}$ test is not rejected are the ones where ${\varvec{\Psi }}$ is non-diagonal and the common factors are either orthogonal or oblique. The first model is also the best one according to the AIC criterion. We thus investigate the solution of such a model.

**Table 3:** Diagonal elements of the scaling matrix **D**. (Standard errors are within parentheses.)

Extraversion (*E*)			Test anxiety (*A*)			Impulsivity (*I*)			Acad. achiev. motiv. (*M*)		
*P*	*T*	*S*	*P*	*T*	*S*	*P*	*T*	*S*	*P*	*T*	*S*
0.81 (0.09)	0.44 (0.12)	0.72 (0.12)	0.79 (0.08)	0.65 (0.10)	0.97 (0.09)	0.72 (0.09)	0.72 (0.08)	0.84 (0.08)	0.43 (0.13)	0.43 (0.12)	0.71 (0.10)

**Table 4:** Estimated factor loading matrix for the traits. (Standard errors are within parentheses.)

	Factor 1	Factor 2	Factor 3
Extraversion (*E*)	1	1	1
Test anxiety (*A*)	$-$ 1.04 (0.56)	$-$ 2.07 (1.26)	$-$ 0.04 (0.09)
Impulsivity (*I*)	2.38 (1.10)	0.36 (0.34)	0.44 (0.21)
Acad. achiev. motiv. (*M*)	$-$ 4.45 (2.56)	$-$ 2.92 (2.03)	0.32 (0.20)

**Table 5:** Estimated factor loading matrix for the methods. (Standard errors are within parentheses.)

	Factor 1	Factor 2	Factor 3
Peer report (*P*)	1	1	1
Teacher rating (*T*)	0.50 (0.14)	1.70 (0.40)	3.56 (0.93)
Self-rating (*S*)	0.44 (0.11)	0.39 (0.15)	1.78 (0.56)

First of all, we report the estimates of the diagonal scaling matrix **D** in Table 3. All these estimates appear to be significant. The estimated factor loadings for the traits and the methods are given in Tables 4 and 5. Note that, without loss of fit, in the estimation process the elements of the first rows of the factor loading matrices for the traits and the methods are constrained to 1. Factor 1 is related to the duality between Impulsivity (with positive sign) versus Academic achievement motivation (with negative sign) mainly measured by Peer report. Factor 2 depends on Test anxiety and Academic achievement motivation (both with negative sign) with respect to the traits and Teacher rating and Peer report (both with positive sign) with respect to the methods. Finally, Factor 3 is essentially related to the measurements by Teacher rating for Extraversion. (All such factor loadings are positive.) The square root of the estimated covariance matrix for the common factors, ${\varvec{\Phi }}^{1/2}$, and of the banded covariance matrix for the unique factors, ${\varvec{\Psi }}^{1/2}$, are given in Tables 6 and 7, respectively. From Table 7, we can see that several off-diagonal elements are rather different from zero. The banded structure of ${\varvec{\Psi }}$ implies differences in the estimated common factors in comparison with the model where ${\varvec{\Psi }}$ is assumed to be diagonal. To clarify this point, we briefly compare the previously described solution with the corresponding one where ${\varvec{\Psi }}$ is diagonal. Although the null hypothesis of the $\chi ^{2}$ test is rejected, such a model is characterized by the lowest BIC. The interpretation is slightly modified due to the loading of Impulsivity which remarkably increases (3.66). The most relevant differences for Factor 2 involve Academic achievement motivation (loading from $-\,2.92$ to $-\,1.35$) and Teacher rating (from 1.70 to 1.26). With respect to Factor 3, we observe differences in the methods. In detail, the loadings of Teacher rating and Self-rating reduce to 2.13 and 1.03, respectively. Obviously, these differences are related to the fact that assuming ${\varvec{\Psi }}$ to be diagonal leads to a model such that the common factors fit some amount of error, i.e., the correlations between the specific factors within the traits. Finally, by looking at the standard errors reported in Tables 3, 4, 5, 6 and 7, we note that in some cases, especially for the variable factor loadings, some estimates are not significantly different from zero. This is quite obvious when 42 parameters are estimated by using only 68 observations.

**Table 6:** Diagonal elements of the square root of the estimated covariance matrix for the common factors ${\varvec{\Phi }}^{1/2}$. (Standard errors are within parentheses.)

Factor 1	Factor 2	Factor 3
$-$ 0.40 (0.16)	0.33 (0.19)	0.56 (0.21)

**Table 7:** Square root of the estimated covariance matrix for the unique factors ${\varvec{\Psi }}^{1/2}$. (Standard errors are within parentheses.)

	*P*				*T*				*D*			
	*E*	*A*	*I*	*M*	*E*	*A*	*I*	*M*	*E*	*A*	*I*	*M*
*P*												
*E*	0.71 (0.23)				0.66 (0.26)				0.24 (0.19)			
*A*		0.86 (0.07)				0.36 (0.14)				0.35 (0.11)		
*I*			0.87 (0.06)				0.48 (0.11)				0.06 (0.17)	
*M*				0.96 (0.19)				$-$ 0.15 (0.59)				$-$ 0.24 (0.47)
*T*												
*E*					0.94 (0.22)				$-$ 0.35 (0.58)			
*A*						0.97 (0.04)				0.26 (0.14)		
*I*							0.99 (0.02)				0.12 (0.15)	
*M*								0.98 (0.05)				0.17 (0.28)
*S*												
*E*									1			
*A*										1		
*I*											1	
*M*												1

## Conclusion and Final Remarks

The structural Parafac model has been discussed in this paper. The focus has been on its major property: uniqueness of factor loading matrices. We have shown that, under mild conditions, such a property, proved only for the component version of the model, holds. In particular, we have shown that it holds even if the specific factors are assumed to be within-variable or within-occasion correlated and the model is modified to become scale invariant. In this respect, a consideration is in order. Our uniqueness proofs are based on Kruskal’s sufficient condition (1977) formulated for the component version of the model. However, this could be substituted with any other sufficient condition that exploits the particular features of the structural model. As an example, in this context, Kruskal’s condition is always applied by having one of the three matrices square and of full rank, i.e., ${\varvec{\Phi }}^{1/2}$. Conditions taking into account such a feature (e.g., Jiang & Sidiropoulos, 2004) probably would lead to more powerful results. Furthermore, we have focused our attention to the structural Parafac model with unconstrained matrices **B** and **C**. However, especially in the component-based version, such matrices and **A** can be constrained (e.g., column-wise orthonormality, linear dependency, nonnegativity, unimodality, symmetry). In general, imposing constraints implies replacing Kruskal’s condition with more relaxed uniqueness conditions. For instance, in case of linear dependency, see Stegeman & Lam (2012). Our results might be improved by using such relaxed conditions.

## References

[CR1] Acar E, Yener B (2009). Unsupervised multiway data analysis: A literature survey. IEEE Transactions on Knowledge and Data Engineering.

[CR2] Adachi K, Trendafilov NT (2019). Some inequalities contrasting principal component and factor analyses solutions. Japanese Journal of Statistics and Data Science.

[CR3] Akaike H (1974). A new look at the statistical model identification. IEEE Transactions on Automatic Control.

[CR4] Akaike H (1987). Factor analysis and AIC. Psychometrika.

[CR5] Anderson, T. W., & Rubin, H. (1956). Statistical inference in factor analysis. In *Proceedings of the third berkeley symposium on mathematical statistics and probability, Volume 5: Contributions to econometrics, industrial research, and psychometry* (pp. 111–150). California: University of California.

[CR6] Bartholomew DJ, Knott M, Moustaki I (2011). Latent variable models and factor analysis: A unified approach.

[CR7] Bentler PM, Lee S-Y (1978). Statistical aspects of a three-mode factor analysis model. Psychometrika.

[CR8] Bentler PM, Lee S-Y (1979). A statistical development of three-mode factor analysis model. British Journal of Mathematical and Statistical Psychology.

[CR9] Bentler PM, McClain J (1976). A multitrait-multimethod analysis of reflection-impulsivity. Child Development.

[CR10] Bentler PM, Poon W-Y, Lee S-Y (1988). Generalized multimode latent variable models: Implementation by standard programs. Computational Statistics and Data Analysis.

[CR11] Bloxom B (1968). A note on invariance in three-mode factor analysis. Psychometrika.

[CR12] Bloxom B, Law HG, Snyder CW, Hattie JA, McDonald RP (1984). Tucker’s three-mode factor analysis. Research methods for multimode data analysis.

[CR13] Bro R (1998). Multi-way analysis in the food industry, models algorithms and applications.

[CR14] Bro R, Sidiropoulos ND, Smilde AK (2002). Maximum likelihood fitting using ordinary least squares algorithms. Journal of Chemometrics.

[CR15] Bro P, Smilde AK (2003). Centering and scaling in component analysis. Journal of Chemometrics.

[CR16] Browne MW (1974). Generalized least squares estimators in the analysis of covariance structures. South African Statistical Journal.

[CR17] Browne MW (1980). Factor analysis of multiple batteries by maximum likelihood. British Journal of Mathematical and Statistical Psychology.

[CR18] Browne MW, Hawkins DM (1982). Covariance structures. Topics in applied multivariate analysis.

[CR19] Browne MW (1984). The decomposition of multitrait-multimethod matrices. British Journal of Mathematical and Statistical Psychology.

[CR20] Browne MW (1984). Asymptotically distribution-free methods for the analysis of covariance structures. British Journal of Mathematical and Statistical Psychology.

[CR21] Carroll JD, Chang JJ (1970). Analysis of individual differences in multidimensional scaling via an $$n$$-way generalization of Eckart–Young decomposition. Psychometrika.

[CR22] De Lathauwer L, De Moor B, Vandewalle J (2000). A multilinear singular value decomposition. SIAM Journal on Matrix Analysis and Applications.

[CR23] Domanov I, De Lathauver L (2013). On the uniqueness of the canonical polyadic decomposition of third-order tensors—Part I: Basic results and uniqueness of one factor matrix. SIAM Journal on Matrix Analysis and Applications.

[CR24] Domanov I, De Lathauver L (2013). On the uniqueness of the canonical polyadic decomposition of third-order tensors—Part II: Uniqueness of the overall decomposition. SIAM Journal on Matrix Analysis and Applications.

[CR25] Eckart C, Young G (1936). The approximation of one matrix by another of lower rank. Psychometrika.

[CR26] Gerard D, Hoff PD (2015). Equivariant minimax dominators of the MLE in the array normal model. Journal of Multivariate Analysis.

[CR27] Giordani P, Kiers HAL (2018). A review of tensor-based methods and their application to hospital care data. Statistics in Medicine.

[CR28] Harshman RA (1970). Foundations of the PARAFAC procedure: Models and conditions for an ‘explanatory’ multi-model factor analysis. UCLA Working Papers in Phonetics.

[CR29] Harshman RA (1972). PARAFAC2: Mathematical and technical notes. UCLA Working Papers in Phonetics.

[CR30] Harshman RA, Lundy ME, Law HG, Snyder CW, Hattie JA, McDonald RP (1984). Data preprocessing and the extended PARAFAC model. Research methods for multimode data analysis.

[CR31] Hoff PD (2016). Equivariant and scale-free Tucker decomposition models. Bayesian Analysis.

[CR32] Jiang T, Sidiropoulos ND (2004). Kruskal’s permutation lemma and the identification of CANDECOMP/PARAFAC and bilinear models with constant modulus constraints. IEEE Transactions on Signal Processing.

[CR33] Kiers HAL (2000). Towards a standardized notation and terminology in multiway analysis. Journal of Chemometrics.

[CR34] Kiers HAL (2006). Properties of and algorithms for fitting three-way component models with offset terms. Psychometrika.

[CR35] Kolda TG, Bader BW (2009). Tensor decompositions and applications. SIAM Review.

[CR36] Kroonenberg PM, De Leeuw J (1980). Principal component analysis of three-mode data by means of alternating least squares algorithms. Psychometrika.

[CR37] Kroonenberg PM, Oort FJ (2003). Three-mode analysis of multi-mode covariance matrices. British Journal of Mathematical and Statistical Psychology.

[CR38] Kroonenberg PM (2008). Applied multiway data analysis.

[CR39] Kruskal JB (1977). Three-way arrays: Rank and uniqueness of trilinear decomposition, with application to arithmetic complexity and statistics. Linear Algebra and Its Applications.

[CR40] Lee S-Y (2007). Structural equation modeling: A Bayesian approach.

[CR41] Lee S-Y, Fong W (1983). A scale invariant model for three-mode factor analysis. British Journal of Mathematical and Statistical Psychology.

[CR42] Mayekawa S (1987). Maximum likelihood solution to the PARAFAC model. Behaviormetrika.

[CR43] Mørup M (2011). Applications of tensor (multi-way array) factorizations and decompositions in data mining. WIREs Data Mining and Knowledge Discovery.

[CR44] Narayanan A (2012). A review of eight software packages for structural equation modeling. The American Statistician.

[CR45] Schwarz GE (1978). Estimating the dimension of a model. Annals of Statistics.

[CR46] Smilde AK, Bro R, Geladi P (2004). Multi-way analysis: Applications in the chemical sciences.

[CR47] Stegeman A (2009). On uniqueness conditions for Candecomp/Parafac and Indscal with full column rank in one mode. Linear Algebra and Its Applications.

[CR48] Stegeman A, Lam TTT (2012). Improved uniqueness conditions for canonical tensor decompositions with linearly dependent loadings. SIAM Journal on Matrix Analysis and Applications.

[CR49] Stegeman A, Lam TTT (2014). Three-mode factor analysis by means of Candecomp/Parafac. Psychometrika.

[CR50] Stegeman A, ten Berge JMF, De Lathauwer L (2006). Sufficient conditions for uniqueness in Candecomp/Parafac and Indscal with random component matrices. Psychometrika.

[CR51] Tucker LR, Harris CW (1963). Implications of factor analysis of three-way matrices for measurement of change. Problems in measuring change.

[CR52] Tucker LR (1966). Some mathematical notes on three-mode factor analysis. Psychometrika.

[CR53] Unkel S, Trendafilov NT (2010). Simultaneous parameter estimation in exploratory factor analysis: An expository review. International Statistical Review.

[CR54] Vega-Montoto L, Gu H, Wentzell PD (2005). Mathematical improvements to maximum likelihood parallel factor analysis: Theory and simulations. Journal of Chemometrics.

[CR55] Vega-Montoto L, Wentzell PD (2003). Maximum likelihood parallel factor analysis (MLPARAFAC). Journal of Chemometrics.

[CR56] Vega-Montoto L, Wentzell PD (2006). Approaching the direct exponential curve resolution algorithm from a maximum likelihood perspective. Analytica Chimica Acta.

